# Predicting the immunological nonresponse to antiretroviral therapy in people living with HIV: a machine learning-based multicenter large-scale study

**DOI:** 10.3389/fcimb.2025.1466655

**Published:** 2025-03-11

**Authors:** Suling Chen, Lixia Zhang, Jingchun Mao, Zhe Qian, Yuanhui Jiang, Xinrui Gao, Mingzhu Tao, Guangyu Liang, Jie Peng, Shaohang Cai

**Affiliations:** ^1^ Department of Infectious Diseases, Nanfang Hospital, Southern Medical University, Guangzhou, China; ^2^ State Key Laboratory of Organ Failure Research, Key Laboratory of Infectious Diseases Research in South China, Ministry of Education, Guangdong Provincial Key Laboratory of Viral Hepatitis Research, Guangdong Provincial Clinical Research Center for Viral Hepatitis, Guangdong Institute of Hepatology, Guangzhou, China; ^3^ Department of Infectious Diseases, The Fifth Affiliated Hospital of Zunyi Medical University, Zhuhai, China; ^4^ Second Department of Elderly Respiratory, Guangdong Provincial People’s Hospital, Guangdong Academy of Medical Sciences, Guangdong Provincial Geriatrics Institute, Southern Medical University, Guangzhou, China

**Keywords:** HIV/AIDS, CD4+ T cell counts, highly active antiretroviral therapy, immune reconstitution, immunological nonresponse

## Abstract

**Background:**

Although highly active antiretroviral therapy (HAART) has greatly enhanced the prognosis for people living with HIV (PLWH), some individuals fail to achieve adequate immune reconstitution, known as immunological nonresponse (INR), which is linked to poor prognosis and higher mortality. However, the early prediction and intervention of INR remains challenging in South China.

**Methods:**

This study included 1,577 PLWH who underwent at least two years of HAART and clinical follow-up between 2017 and 2022 at two major tertiary hospitals in South China. We utilized logistic multivariate regression to identify independent predictors of INR and employed restricted cubic splines (RCS) for nonlinear analysis. We also developed several machine-learning models, assessing their performance using internal and external datasets to generate receiver operating characteristic (ROC) curves, calibration curves, and decision curve analysis (DCA). The best-performing model was further interpreted using Shapley additive explanations (SHAP) values.

**Results:**

Independent predictors of INR included baseline, 6-month and 12-month CD4+ T cell counts, baseline hemoglobin, and 6-month hemoglobin levels. RCS analysis highlighted significant nonlinear relationships between baseline CD4+ T cells, 12-month CD4+ T cells and baseline hemoglobin with INR. The Random Forest model demonstrated superior predictive accuracy, with ROC areas of 0.866, 0.943, and 0.897 across the datasets. Calibration was robust, with Brier scores of 0.136, 0.102, and 0.126. SHAP values indicated that early CD4+T cell counts and CD4/CD8 ratio were crucial in predicting INR.

**Conclusions:**

This study introduces the random forest model to predict incomplete immune reconstitution in PLWH, which can significantly assist clinicians in the early prediction and intervention of INR among PLWH.

## 1 Introduction

Highly active antiretroviral therapy (HAART) is regarded as the most efficacious approach to treating HIV infection, effectively suppressing viral replication and facilitating immune reconstitution (Yan et al., 2023). However, there is increasing evidence that poor immune reconstitution remains a common issue in clinical practice, with prevalence rates potentially exceeding 10-40% (Ma et al., 2024; Yang et al., 2020; Liu et al., 2024). Despite complete viral suppression by HAART, people living with HIV (PLWH) who experience immune non-response (INR) face increased risks of both AIDS-defining and non-AIDS-defining illnesses (García et al., 2004; Achhra et al., 2010; Trickey et al., 2017). Consequently, clinical guidelines recommend using clinical immunological monitoring as an alternative biomarker of treatment response to identify non-responders to HAART early (Deeks et al., 2015; AIDS and Hepatitis C Professional Group et al., 2021). Subsequently, the recovery of CD4+ T cell counts post-HAART has gradually become one of the predictors of clinical prognosis in PLWH (Committee TUCHC (CHIC) SS, 2007; Guiguet et al., 2009; Pu and Wu, 2024).

Numerous cohort studies have evaluated factors associated with CD4+ T cell recovery post-HAART, identifying that older age, lower baseline CD4+ T cell counts, higher baseline HIV RNA levels, reduced thymic function, increased T cell activation during treatment, and detectable viremia are all linked to poorer CD4+ T cell recovery (Kaufmann et al., 2005; Gazzola et al., 2009; Boatman et al., 2019; Yang et al., 2020). In recent years, a variety of mathematical models have been developed for the prevention and treatment of HIV/AIDS (Nah et al., 2017; Mutoh et al., 2018; Wang et al., 2021; Li et al., 2024), which have provided theoretical guidance and recommendations for HIV treatment. However, the current models predominantly rely on traditional linear approaches such as logistic regression (Wang et al., 2024). This gap suggests a need for more sophisticated modeling techniques that can integrate a broader range of biological markers and dynamic changes over time to enhance the prediction and management of HIV treatment outcomes.

In this study, we aimed to identify risk factors for INR among PLWH in South China who have been treated with standard HAART for at least 2 years. The objective is to develop machine learning predictive models that utilize multiple clinical indicators from baseline, 6 months, and 12 months to predict whether they will experience INR after two years of HAART. This model will assist clinicians in timely predicting immune responses and implementing interventions to enhance immune function. Additionally, the calibration and diagnostic capabilities of the machine learning models were evaluated in both internal and external validation sets.

## 2 Methods

### 2.1 Study design and participants inclusion and exclusion criteria

**Figure 1:**
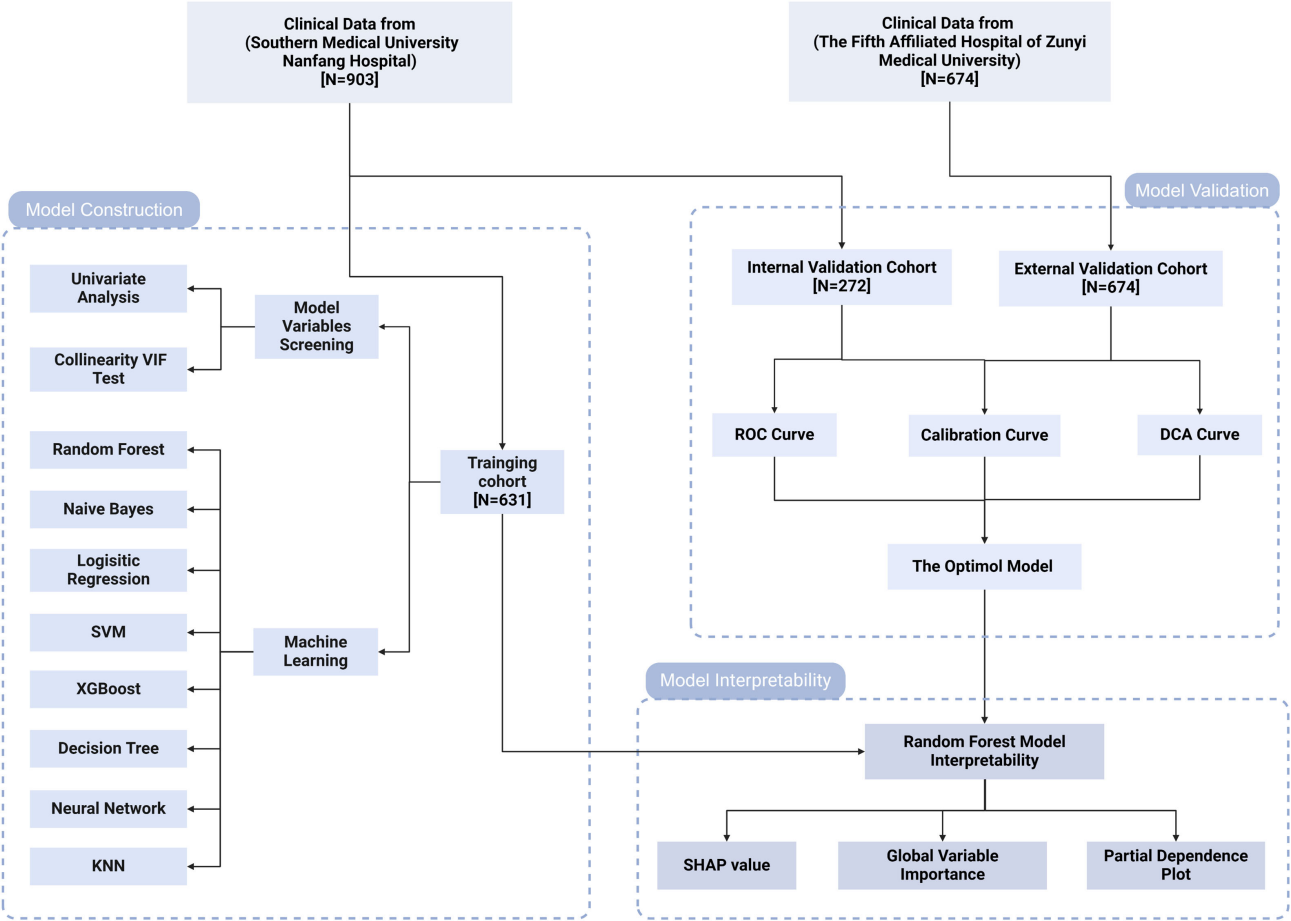
Study flow diagram. VIF, variance inflation factor; SVM, support vector machine; KNN, k-nearest neighbors; ROC, receiver operating characteristic; DCA, decision curve analysis; SHAP, SHapley Additive exPlanations.

This study is based on the follow-up cohorts of PLWH at Nanfang Hospital and the Fifth Hospital of Zunyi, where participants have been undergoing long-term treatment and regular follow-ups at HIV clinics. A total of 1577 participants were enrolled based on defined inclusion and exclusion criteria. The inclusion criteria were: 1) a baseline CD4+ T cell counts of less than 350 cells/μL at the initiation of HAART, with continuous follow-up for 2 years, and two HIV RNA measurements of less than 50 copies/mL; 2) age 18 years or older, with complete baseline, 6-month, 12-month, and 24-month CD4+ T cell counts. The exclusion criteria included: 1) poor treatment adherence or a history of treatment interruption; 2) concurrent malignancy or long-term use of immunosuppressive medications; and 3) incomplete clinical data. As illustrated in **Figure 1**, the cohort from Nanfang Hospital was divided into a training set and an internal validation set in a 7:3 ratio, while the cohort from the Fifth Hospital of Zunyi was designated as the external validation set.

### 2.2 Ethics approval and consent to participate

The research received approval from the Institutional Ethics Committee of Nanfang Hospital (study identifier: NFEC-2021-448) and adhered to the Helsinki Declaration of 1964, along with its subsequent updates. Informed consent was obtained from all participants.

### 2.3 Data collection and definition

We systematically collected demographic and clinical parameters of participants including age, gender, HAART regimens, HBsAg positivity, anti-HCV positivity, HIV viral load, and laboratory measurements at baseline, 6 months, 12 months, and 24 months into treatment. These measurements encompassed CD4+ T cell counts, CD8+ T cell counts, CD4/CD8 ratios, Platelet (PLT), creatinine (CR), hemoglobin (HGB), white blood cell count (WBC), aspartate aminotransferase (AST), alanine aminotransferase (ALT), triglycerides (TG), total cholesterol (CHOL), and fasting plasma glucose (FPG). The aforementioned data were obtained from clinical records or databases.

Currently, there is no universally accepted definition for immune reconstitution failure. In this study, INR was defined as having two consecutive HIV RNA measurements <50 copies/mL after two years of HAART, still maintaining a CD4+ T lymphocyte count of <350 cells/µL (Cuzin et al., 2007; Gunda et al., 2017).

### 2.4 Construction, evaluation, and interpretation of predictive models

In this study, variables from the training set that demonstrated significance at a p-value <0.05 in univariate analysis were included in the model construction. We employed several machine learning algorithms to predict INR classification, including the Logistic Regression Model (LRM), Random Forest (RF), XGBoost, Support Vector Machine (SVM), Naive Bayes, Decision Trees, neural network, and k-nearest Neighbors (KNN). To prevent overfitting and enhance the generalizability of the models, a 10-fold cross-validation method was employed for model evaluation, with iterative refinements through repeated trials.

To further assess and compare the predictive performance of these models, we constructed receiver operating characteristic (ROC) curves and determined the area under the ROC curve (AUC). An AUC value closer to 1 indicates better predictive performance. Additionally, we utilized calibration curves to evaluate the consistency between the observed and predicted risks. The more the calibration curve of the model aligns with the 45 - degree line, and the closer the value of the Brier score is to 0, the more the predicted probability matches the observed event incidence. Furthermore, decision curve analysis (DCA) was used to evaluate the clinical utility of the models. By comparing the net benefits of the model with two default strategies (treating all or none), DCA provides insights into the clinical value of the models.

To improve the interpretability of machine learning models, which are often regarded as “black box” models due to their complex and opaque decision-making processes, we applied Shapley Additive Explanations (SHAP) analysis. SHAP is a cooperative game theory-based approach that quantifies each feature’s contribution by assessing its influence on model predictions. A SHAP value greater than 0 indicates a positive contribution of the feature to the prediction, while a value less than 0 indicates a negative contribution. The larger the SHAP value, the greater the feature’s influence on the prediction. In our study, we visualized these contributions using importance ranking charts, which highlight the relative weight of each feature in influencing the outcome. Additionally, we employed partial dependence plots to demonstrate how each feature affects the predicted results, illustrating the relationship between individual features and the model’s output while considering the influence of other variables.

### 2.5 Statistical analysis

In our analysis, datasets that conformed to a normal distribution were described using the mean ± standard deviation, and comparisons between two groups were conducted using Student’s t-test. For datasets that were non-normally distributed, comparisons were made based on the median and interquartile range, with the Mann-Whitney U test applied for statistical evaluation. Categorical variables were summarized as frequencies and percentages and analyzed using either the chi-square test or Fisher’s exact test, as appropriate. Independent risk factors for INR were identified through univariate and multivariate logistic regression analysis. To evaluate the dose-response relationship between continuous variables and INR, we employed restricted cubic splines (RCS). This method enables the visualization and quantification of potential non-linear associations, and by analyzing the shape of the dose-response curve, we can identify critical thresholds where the relationship between the predictor and the outcome changes. It is important to note that all aspects of data analysis and graphical representation were performed using R version 4.2.1. All tests conducted in this study were two-tailed, and a p-value <0.05 was considered statistically significant.

## 3 Result

### 3.1 Baseline characteristics and follow-up data changes in PLWH

In the longitudinal cohort study of PLWH to predict the risk of INR during follow-up, we retrospectively included 903 PLWH from Nanfang Hospital and 674 PLWH from the Fifth Hospital of Zunyi University, who had been under treatment for more than two years. These cohorts served as the internal and external datasets, respectively. As shown in **Table 1**, Nanfang Hospital enrolled 903 participants, with 532 achieving immune response (IR) and 371 not achieving IR, while the Fifth Hospital of Zunyi University included 674 participants, with 408 in the IR group and 266 in the INR group. In both cohorts, the INR group exhibited significantly higher ages and viral loads compared to the IR group, while CD4+ T cell counts were notably lower in the INR group. There were no significant differences between the two groups in terms of gender, HAART regimens, and the prevalence of baseline HBsAg and anti-HCV.

**Table 1:** The baseline clinical characteristics of the internal and external datasets.

Characteristics	Nanfang Hospital, N= 903			The Fifth Affiliated Hospital of Zunyi Medical University, N = 674		
	IR, N = 532	INR, N = 371	P value	IR, N = 408	INR, N = 266	P value
Age, years	31.00 [24.75, 41.25]	35.00 [27.50, 46.50]	<0.001	30.00 [25.00, 40.00]	36.50 [29.00, 46.00]	<0.001
Gender			0.064			0.578
Female	41 (7.71%)	42 (11.32%)		61 (14.95%)	44 (16.54%)	
Male	491 (92.29%)	329 (88.68%)		347 (85.05%)	222 (83.46%)	
HAART Regimen			0.225			0.039
INSTI based	140 (26.32%)	116 (31.27%)		29 (7.11%)	33 (12.41%)	
NNRTI based	380 (71.43%)	245 (66.04%)		342 (83.82%)	216 (81.20%)	
PI based	12 (2.26%)	10 (2.70%)		37 (9.07%)	17 (6.39%)	
Baseline HBsAg			0.179			0.360
HBsAg negative	472 (88.72%)	318 (85.71%)		368 (90.20%)	234 (87.97%)	
HBsAg positive	60 (11.28%)	53 (14.29%)		40 (9.80%)	32 (12.03%)	
Baseline AntiHCV			0.744			0.811
AntiHCV negative	526 (98.87%)	368 (99.19%)		397 (97.30%)	258 (96.99%)	
AntiHCV positive	6 (1.13%)	(0.81%)		11 (2.70%)	8 (3.01%)	
Baseline HIV load, log10(copies/ml)	4.31 [3.74, 4.74]	4.50 [3.87, 4.90]	0.003	4.55 [4.14, 4.98]	4.84 [4.37, 5.17]	<0.001
Baseline CD4+T cells, cells/μl	252.00 [199.75, 298.00]	122.00 [59.00, 191.50]	<0.001	249.00 [183.00, 301.00]	121.50 [34.25, 192.75]	<0.001

HAART, highly active antiretroviral therapy; INSTI, integrase strand transfer inhibitor; NNRTI, non-nucleoside reverse transcriptase inhibitor; PI, protease inhibitor; HBsAg, hepatitis B surface antigen; AntiHCV, anti-hepatitis C virus antibody.

**Figure 2:**
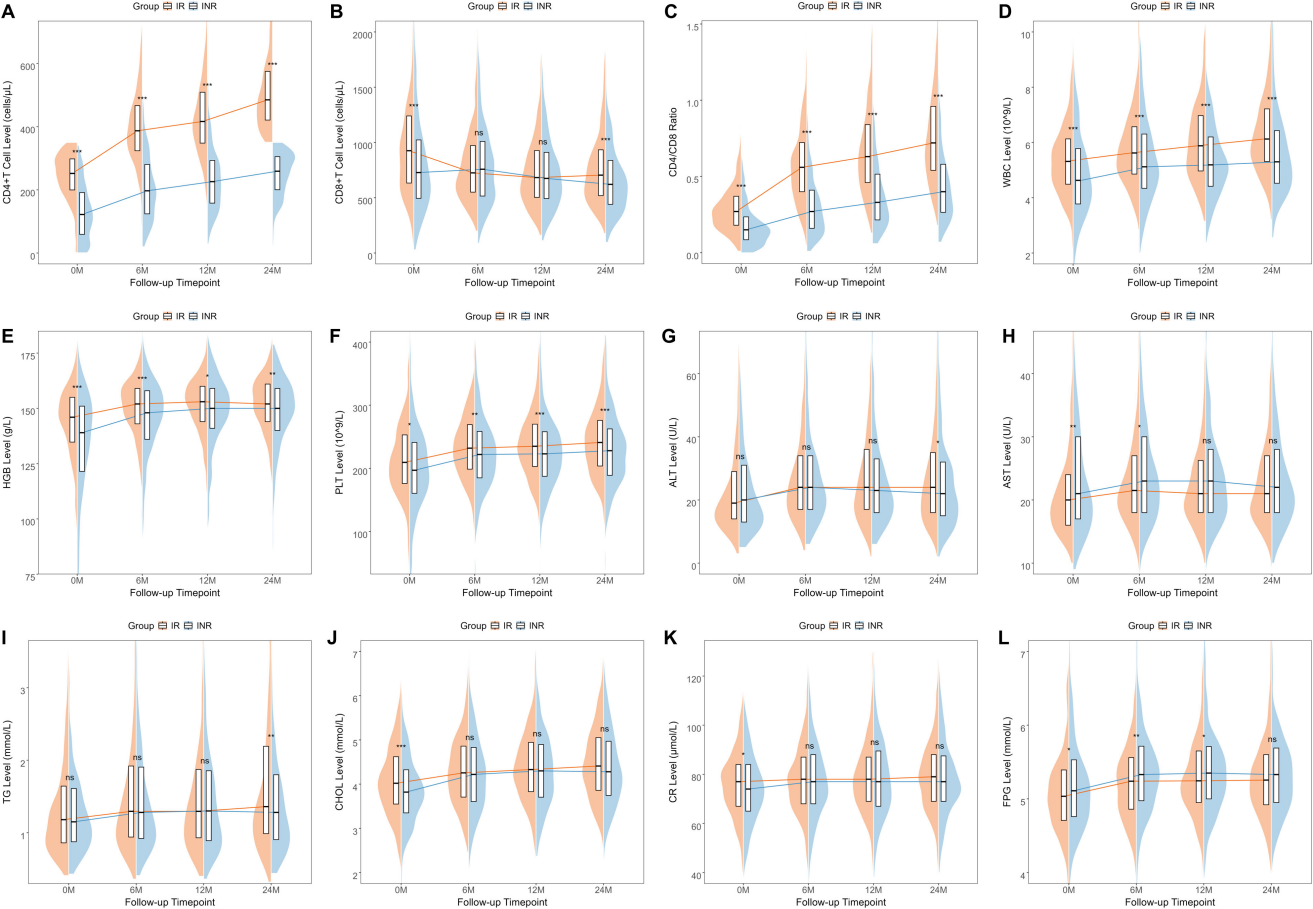
The changes in the clinical characteristics of PLWH within the internal dataset across four follow-up points The changes in various clinical characteristics at different follow-up time points including CD4+T cells **(A)**, CD8+T cells **(B)**, CD4/CD8 ratio **(C)**, WBC **(D)**, HGB **(E)**, PLT **(F)**, ALT **(G)**, AST **(H)**, TG **(I)**, CHOL **(J)**, CR **(K)**, and FPG **(L)**. WBC, white blood cells; HGB, hemoglobin; PLT, platelets; ALT, alanine aminotransferase; AST, aspartate aminotransferase; TG, triglycerides; CHOL, cholesterol; CR, creatinine; FPG, fasting plasma glucose.

We visualized the clinical characteristics of PLWH at each follow-up point using line graphs (**Figure 2**) and compared the levels between the IR group and the INR group. We observed that at each follow-up point, the IR group exhibited higher levels of CD4+ T cells, CD4/CD8 ratio, WBC counts, HGB levels, and PLT levels compared to the INR group. However, differences in CD8+ T cells, liver function markers such as ALT and AST, lipid levels including TG and CHOL, renal function as indicated by CR, and FPG were only present at certain follow-up points. A similar analysis was conducted in the external dataset (**Supplementary Figure 1**), and the results were consistent. The only exception was that the CD8+ T cell levels were also higher in the IR group compared to the INR group.

### 3.2 Independent risk factors associated with poor immune response in PLWH

**Figure 3:**
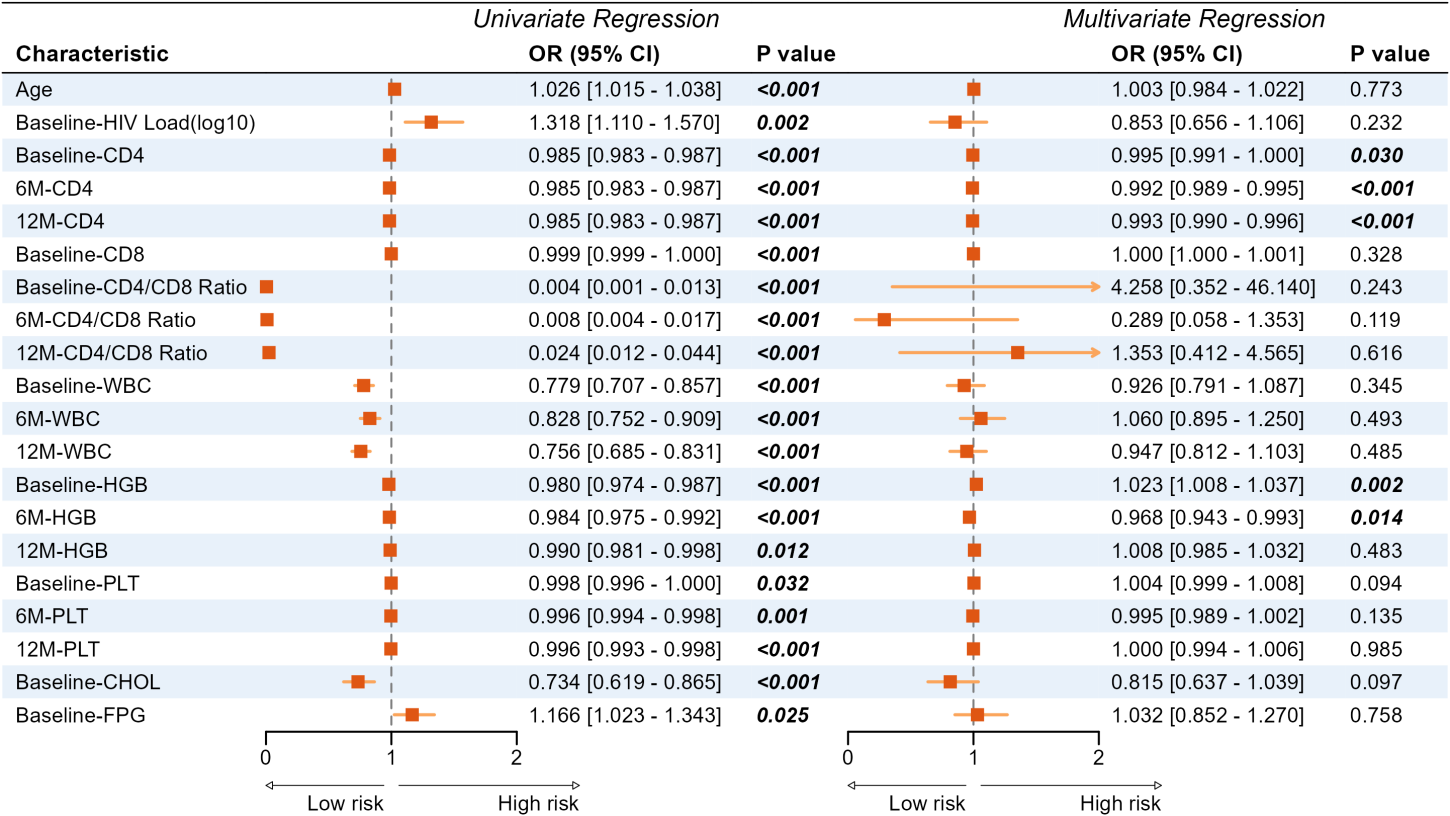
Univariate and multivariate analysis of immune non-reconstitution. WBC, white blood cells; HGB, hemoglobin; CHOL, cholesterol; FPG, fasting plasma glucose; PLT, platelets.

To investigate the factors influencing INR, we conducted a univariate logistic analysis that identified 20 significant variables (**Figure 3**). Given the potential for multicollinearity among these variables, we conducted a collinearity test on variables with a p-value < 0.05 from the logistic univariate analysis by calculating the variance inflation factor (VIF) (**Supplementary Figure 2**). Since all parameters had a VIF value less than 10, all were included in the multivariate analysis and identified independent factors for INR as Baseline-CD4 (OR = 0.995, P = 0.030), 6M-CD4 (OR = 0.992, P < 0.001), 12M-CD4 (OR = 0.993, P < 0.001), Baseline-HGB (OR = 1.023, P = 0.002), and 6M-HGB (OR = 0.968, P = 0.014).

To further analyze the relationship between baseline parameters and INR, we conducted the same analysis and found that in the multivariate analysis (**Supplementary Figure 3**), age (OR = 1.021, P = 0.010), HIV load (OR = 0.725, P = 0.009), baseline CD4 (OR = 0.983, P < 0.001), baseline WBC (OR = 0.842, P = 0.008) and baseline HGB (OR = 1.012, P = 0.014) were independently associated with INR.

### 3.3 Dose-response relationship between 6M-CD4, 12M-CD4, baseline-HGB, 6M-HGB and INR

**Figure 4:**
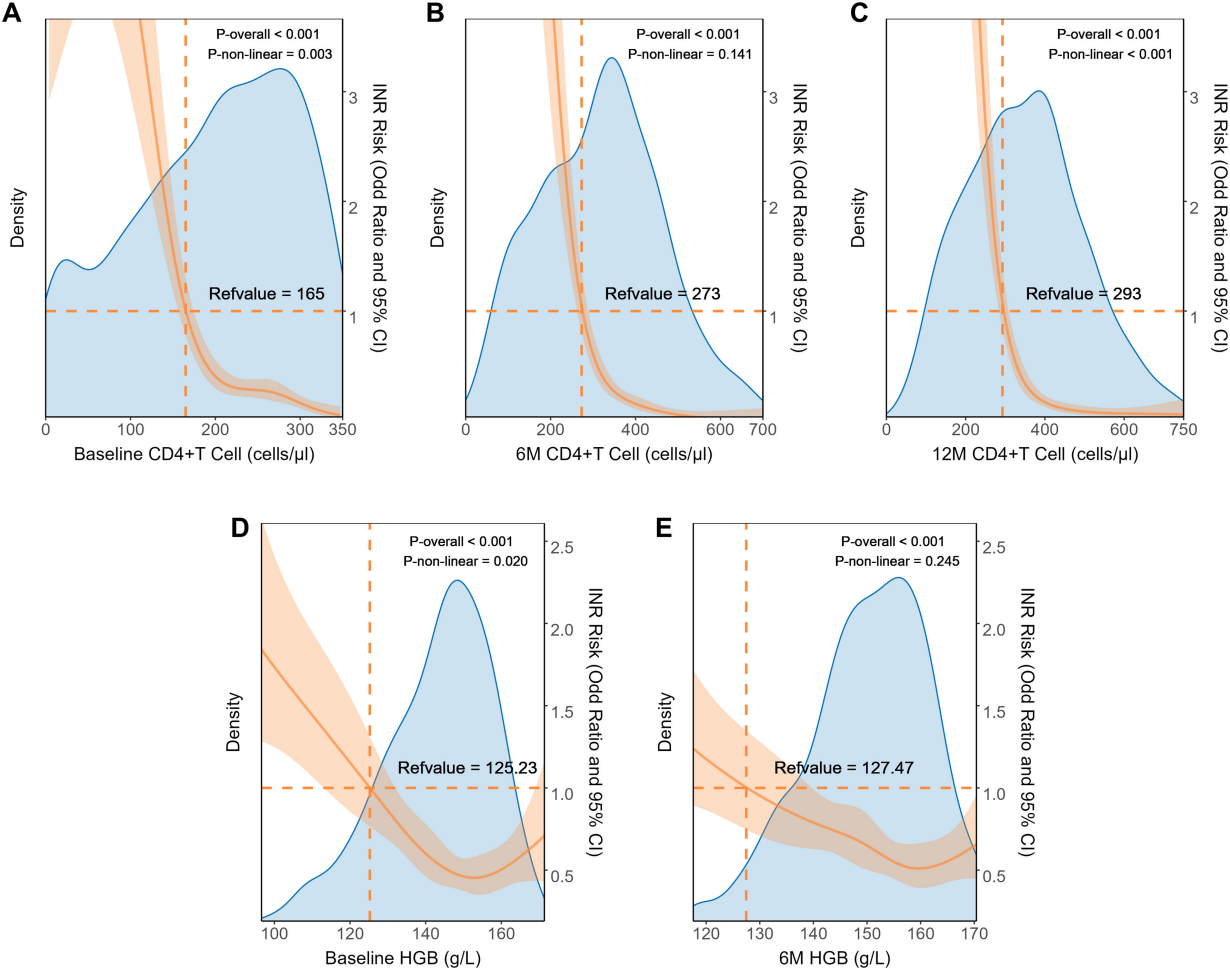
Dose relationship between clinical characteristics and INR in internal dataset. The restricted cubic splines of the association between INR prevalence and clinical parameters including Baseline CD4+T cell **(A)**, 6M CD4+T cell **(B)**, 12M CD4+T cell **(C)**, baseline HGB **(D)**, and 6M-HGB **(E)**. HGB, hemoglobin.

Through RCS analysis, we further investigated the relationship between independent factors and INR incidence (**Figure 4**). We observed that 6M-CD4 and 6M-HGB showed a linear relationship with INR (overall p<0.05, nonlinearity p>0.05), with threshold concentrations of 273 cells/μL and 127.47 g/L, respectively. Conversely, a nonlinear relationship was evident between Baseline-CD4, 12M-CD4, Baseline-HGB, and INR (overall p<0.05, nonlinearity p<0.05). The risk of INR rapidly increased when Baseline-CD4 was below 165 cells/μl, 12M-CD4 was below 293 cells/μl, and Baseline-HGB was less than 125.23 g/L.

### 3.4 Model construction and verification

We divided the internal dataset into a training set for model construction and an internal validation set following a 7:3 split, while the external dataset served as the models’ external validation set. We compared the baseline clinical characteristics across the three datasets (**Supplementary Table 1**). The median age of PLWH in all three datasets was 32 years old, and the proportion of INR was similar across the datasets. Notably, the external validation set had a higher proportion of female PLWH and a lower proportion using INSTI-based treatment regimens.

**Figure 5:**
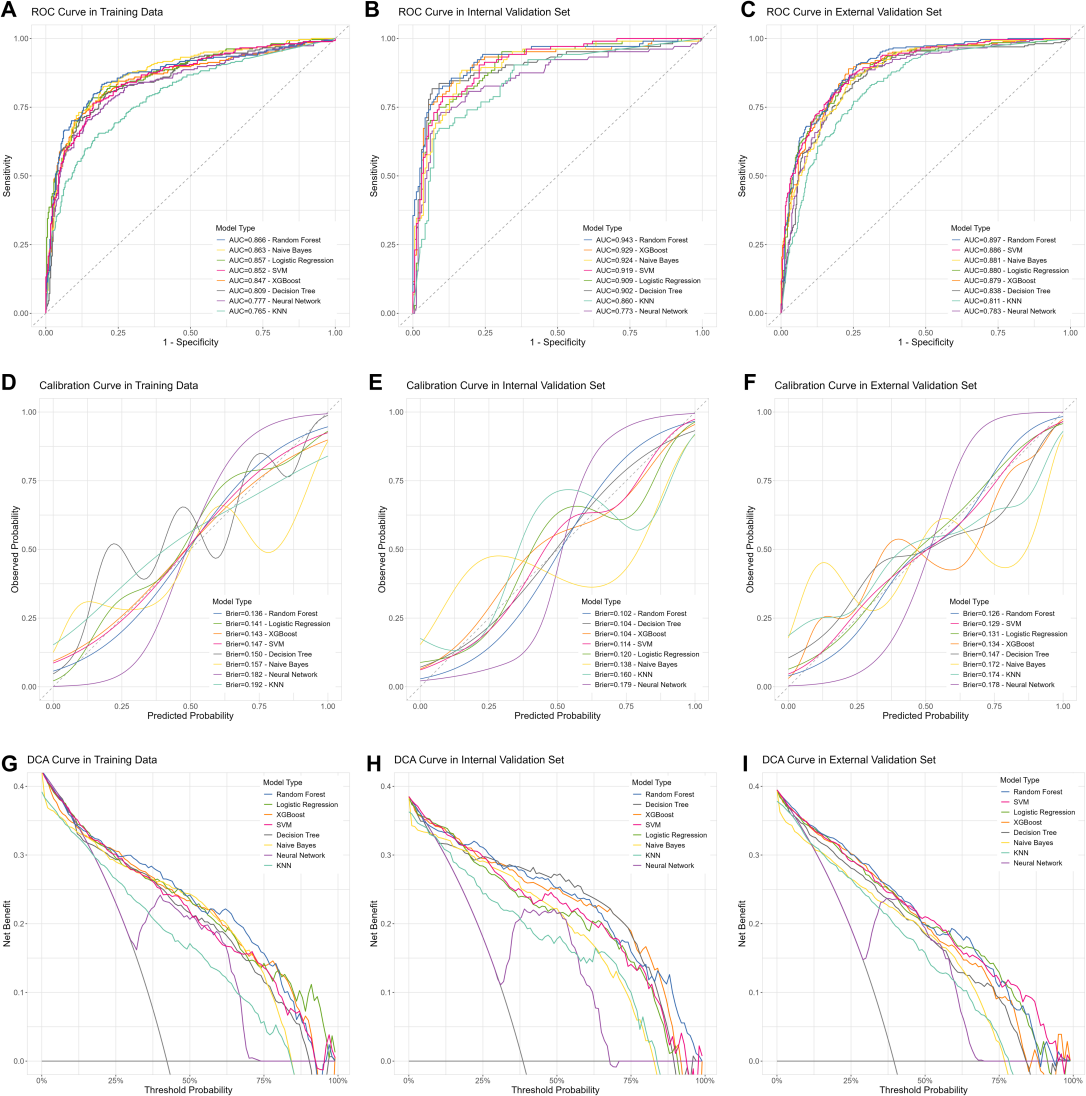
The machine learning models construction and performance evaluation. **(A-C)** ROC curves of models in the training, internal validation, and external validation cohorts. **(D-F)** Calibration plots of models in the training, internal validation, and external validation cohorts. **(G-I)** DCA curves of models in the training, internal validation, and external validation cohorts. SVM, support vector machine; XGBoost, extreme gradient boosting; KNN, k-nearest neighbors.

Subsequently, we incorporated significant variables in the univariate analysis in **Figure 3** into model construction, including Baseline and 6-month/12-month CD4+ T cells, CD4/CD8 ratio, WBC, HGB, PLT and etc. Using these variables, we developed eight predictive models employing machine learning methods. We then validated the stability and generalizability of these eight models across the training, internal, and external validation sets. Ultimately, the RF model exhibited the best clinical predictive performance across all datasets, with AUROC values of 0.866, 0.943, and 0.897, respectively (**Figures 5A–C**). In terms of calibration, the RF model outperformed other models in all three datasets, with Brier scores of 0.136, 0.102, and 0.126 (**Figures 5D–F**). In clinical utility assessment, the DCA curves of the RF model were consistently higher than the “treat all” and most other model lines across the majority of threshold probabilities, indicating significant clinical application value (**Figures 5G–I**).

### 3.5 Interpretability of the optimal model

**Figure 6:**
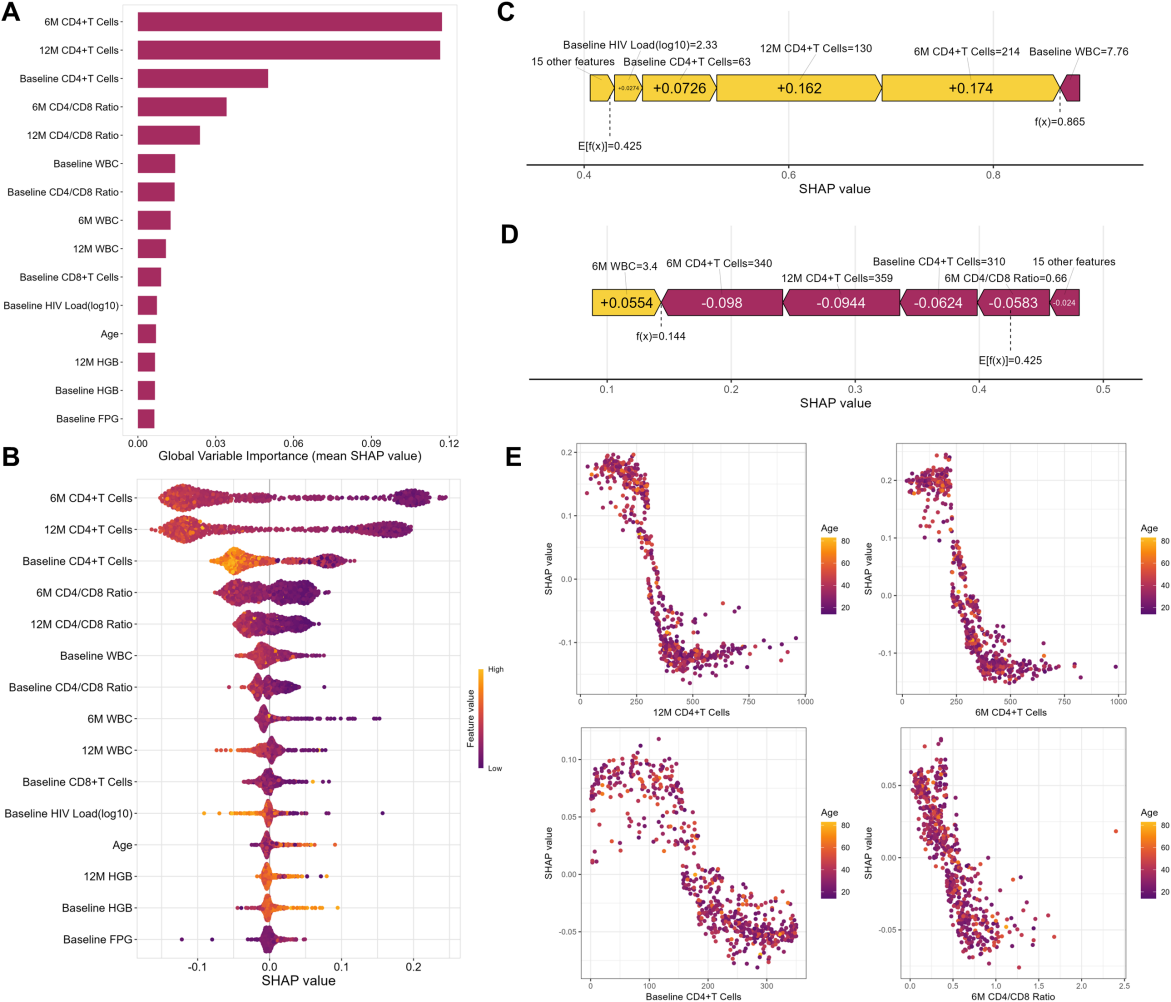
Random Forest Model Interpretability. **(A)** The Feature-ranking plot of the Random Forest Model for predicting INR in PLWH. **(B)** The mean SHAP value of the Random Forest model for predicting INR in PLWH. **(C)** The force plot of the Random Forest model example with an INR PLWH. **(D)** The force plot of Random Forest model example with an IR PLWH. **(E)** The partial dependence plot between SHAP value and top 4 important features. WBC, white blood cells; HGB, hemoglobin; PLT, platelets.

Given the RF model’s outstanding predictive capability across both internal and external validation datasets, we ultimately designated it as the best-performing model. To clarify the clinical relevance of specific features, this research quantified their importance using SHAP values. The variables were prioritized by their impact on predicting INR risk (**Figure 6A**), identifying the top five predictors in PLWH after two years of HAART as 6-month CD4+ T cells, 12-month CD4+ T cells, baseline CD4+ T cells, 6-month CD4/CD8 ratio, and 12-month CD4/CD8 ratio. Consequently, CD4+ T cell counts measured between 6 and 12 months post-treatment are critical for assessing immune reconstitution.

Through the summary plot (**Figure 6B**), we detailed the positive and negative relationships between features and outcomes, finding that higher CD4+T cell counts were associated with a lower probability of INR, and older age correlated with a higher probability of INR. Subsequently, we illustrated the impact of model variables in predictions for an example of PLWH with IR and INR respectively (**Figures 6C, D**). Finally, we generated a partial dependence plot (**Figure 6E**). Specifically, the critical threshold for CD4+ T cell counts was observed around 350 cells/µL at 12 months, 250 cells/µL at 6 months, and 150 cells/µL at baseline. For the 6-month CD4/CD8 ratio, maintaining a value near 0.5 was associated with minimizing INR risk. When the parameter values fall below these critical thresholds, the risk of INR increases. Nevertheless, it is noteworthy that the partial dependence analysis did not detect significant correlations between variables and age.

## 4 Discussion

In this study, we collected data from 1577 PLWH who received at least two years of HAART from two centers. On one hand, we analyzed the changes in clinical parameters at different follow-up points and identified independent risk factors for INR using univariate and multivariate logistic regression. On the other hand, we systematically constructed machine learning predictive models using dataset from Nanfang Hospital, which was further validated and assessed for sensitivity, specificity, and calibration using internal and external datasets. Our findings indicate that the RF model emerged as the best predictor for INR. To our knowledge, this was the first machine learning predictive model specifically developed to predict the occurrence of INR among PLWH in South China. This model not only provides a valuable tool for clinical decision-making but also enhances our understanding of the dynamics and predictors of immune recovery in this population.

Machine learning’s capability to identify high-dimensional nonlinear relationships among clinical features for outcome prediction has been extensively applied in the field of HIV/AIDS research (Rivero-Juárez et al., 2020; Mazrouee et al., 2021; He et al., 2022; Huang et al., 2023). For example, researchers have utilized machine learning methods on electronic health records (EHR) data to precisely identify the burden of comorbidities in PLWH (Yang et al., 2021). In recent years, traditional linear models have been used to predict INR (Gunda et al., 2017; Li et al., 2019; Zhang et al., 2023), and these models have provided auxiliary value in specific clinical practices. Unlike previous studies on INR prediction, this research included a comprehensive set of variables such as liver and kidney functions, lipid and glucose levels, and considers clinical indicators from multiple follow-up points. A machine learning model was constructed, taking into account not only these diverse clinical indicators but also ensuring rigorous internal and external validation of the model. This comprehensive approach enhances the predictive accuracy and reliability of the model, thereby making a significant contribution to clinical decision-making and the management of PLWH.

In the line graphs, we observed that the levels of WBC, HGB, and PLT were significantly higher in the IR group, and multivariate logistic regression analysis indicated that baseline and 6-month HGB levels are independent risk factors for INR. Hematological alterations are prevalent complications in individuals with HIV/AIDS, linked to reduced quality of life and higher mortality rates (Koka and Reddy, 2004; Firnhaber et al., 2010; Shen et al., 2015). Both direct and indirect influences of HIV infection on hematopoietic progenitor cells disturb bone marrow equilibrium and affect the proliferation and differentiation of cells in hematopoiesis, mainly leading to anemia and thrombocytopenia in peripheral blood (Huibers et al., 2020; Tsukamoto, 2020). Moreover, studies have shown that the improvement in CD4+ T cell counts following HAART leads to a decreased prevalence of cytopenias in PLWH, suggesting that HIV-related cytopenias are driven by HIV infection and immune suppression (Choi et al., 2011; Woldeamanuel and Wondimu, 2018). Therefore, this study not only reaffirms the connection between anemia and cytopenias with low CD4+T cell counts but also highlights the predictive value of thrombocytopenia and anemia in PLWH for INR. Considering that anemia and thrombocytopenia are treatable conditions associated with higher mortality rates in PLWH, it is essential to monitor blood cell count changes throughout HIV infection. This monitoring helps identify the onset of these hematological disorders and enables the implementation of vital clinical interventions to avert complications.

To improve the interpretability of the model prediction process, we utilized SHAP values to quantify the impact of each variable on the model's predictions. The results indicated that the CD4+T cell counts at 6M and 12M were crucial factors affecting the occurrence of INR among PLWH. Previous research has frequently reported that baseline CD4+T cell counts was an effective predictor for INR (Rb-Silva et al., 2019; Bayarsaikhan et al., 2021), with studies suggesting that a baseline CD4+T cell counts ≥200 cells/mm (Ma et al., 2024) was independently associated with inconsistent immune response development in multivariate analysis (Muzah et al., 2012). However, this study highlights that, compared to baseline CD4 levels, the CD4+T cell counts at 6M and 12M require more attention. This shift in focus suggests a dynamic approach to monitoring immune recovery, emphasizing the importance of ongoing evaluation beyond initial treatment phases.

It’s noteworthy that after interpreting the RF model using SHAP, we found that CD4+T cell levels and the CD4/CD8 ratio remained the most influential factors in the model. However, earlier research has shown that older age could contribute to insufficient CD4+ T-cell recovery in PLWH, indicating that age can substantially affect the long-term restoration of CD4+ T cells (Burgos et al., 2022; Chen et al., 2022). Additionally, research has included the age at the initiation of HAART in the logistic prediction model for INR (Zhang et al., 2023). Although age was a recognized factor in predicting INR, the partial dependence plot from the partial correlation analysis did not show a clear distributional association between age and CD4+ T cell counts, which might suggest more complex underlying relationships that are influenced by other factors included in the model. Machine learning models, especially those like RF, can capture complex, nonlinear interactions that might not be evident or are assumed away in traditional linear models.

The occurrence of INR is closely associated with cytokine dysregulation (Vos et al., 2024). Chronic inflammation induced by HIV infection can lead to sustained elevations of IL-6 and TNF-α, which impair bone marrow function and suppress hematopoiesis, resulting in reduced T cell production (Huibers et al., 2020; Wan et al., 2023). This process may contribute to anemia and thrombocytopenia, further hindering immune recovery. Additionally, individuals with INR exhibit elevated levels of immunosuppressive cytokines, such as IL-10 and TGF-β, which inhibit T cell proliferation (Zicari et al., 2019). Simultaneously, overexpression of PD-1 on CD4+ T cells promotes immune exhaustion, leading to limited proliferation and increased apoptosis (Zhang et al., 2021). In this study, CD4+ T cell counts were identified as significant predictors of INR, suggesting that chronic inflammation and T cell exhaustion may be potential mechanisms contributing to INR development.

Our study possesses significant strengths. We have constructed machine learning predictive models for early identification of INR in PLWH, integrating multiple clinical indicators from baseline, 6-month, and 12-month follow-up points. The internal and external validations of the model have demonstrated its stability. Furthermore, the parameters used in the model are commonly available in standard clinical settings, requiring no additional measurements. This will assist clinicians in timely predicting immune responses and implementing interventions. Despite these strengths, we acknowledge some constraints in our research. To begin with, its retrospective nature may be affected by inherent drawbacks related to the study design. Additionally, as the study population is exclusively from South China, this raises uncertainties regarding the applicability and generalizability of our proposed predictive model to other populations or ethnic groups. Furthermore, due to limitations in time, resources, and study design, our research lacks mechanistic investigations like cytokine analysis, which could have provided further insights into the immune responses differentiating between responders and non-responders. These limitations highlight areas for future research to expand the model’s robustness and ensure its efficacy across diverse demographic settings.

## 5 Conclusion

This study demonstrates that the Random Forest model has good performance in predicting the risk of INR among PLWH, facilitating early identification and intervention for INR in clinical settings.

## Data Availability

The raw data supporting the conclusions of this article will be made available by the authors, without undue reservation.
