# Supplementary material for: Predicting the immunological nonresponse to antiretroviral therapy in people living with HIV: a machine learning-based multicenter large-scale study
Source: Front Cell Infect Microbiol. 2025 Mar 11;15:1466655. doi: 10.3389/fcimb.2025.1466655 (PMC11933112; doi:10.3389/fcimb.2025.1466655)
Supplement: Supplementary file 1 [file DataSheet1.pdf]

Supplementary Material

Predicting the Immunological Nonresponse to Antiretroviral Therapy in People Living with HIV: A Machine Learning-Based Multicenter Large-Scale Study

Suling Chen, Lixia Zhang, Jingchun Mao, Zhe Qian, Yuanhui Jiang, Xinrui Gao, Mingzhu Tao, Guangyu Liang, Jie Peng, Shaohang Cai

Supplementary Table 1. The clinical characteristics of the training set, internal validation set, and external validation set

Characteristics	Training, N = 631	Internal Validation, N = 272	External Validation, N = 674
Age, years	32.00 [26.00, 43.00]	32.00 [25.00, 43.00]	32.00 [26.00, 43.00]
Gender			
Female	60 (9.51%)	23 (8.46%)	105 (15.58%)
Male	571 (90.49%)	249 (91.54%)	569 (84.42%)
Group			
IR	364 (57.69%)	168 (61.76%)	408 (60.53%)
INR	267 (42.31%)	104 (38.24%)	266 (39.47%)
HAART Regimen			
INSTI based	131 (20.76%)	125 (45.96%)	62 (9.20%)
NNRTI based	483 (76.55%)	142 (52.21%)	558 (82.79%)
PI based	17 (2.69%)	5 (1.84%)	54 (8.01%)
Baseline HBsAg			
Negative	554 (87.80%)	236 (86.76%)	602 (89.32%)
Positive	77 (12.20%)	36 (13.24%)	72 (10.68%)
Baseline AntiHCV			
Negative	627 (99.37%)	267 (98.16%)	655 (97.18%)
Positive	4 (0.63%)	5 (1.84%)	19 (2.82%)
Baseline CD4+T cell, cells/μl	210.00 [130.00, 278.50]	211.00 [106.75, 280.00]	200.00 [102.50, 271.50]
Baseline CD8+T cell, cells/μl	835.00 [571.50, 1,172.00]	848.50 [561.75, 1165.75]	761.50 [520.00, 964.75]
Baseline CD4/CD8 ratio	0.22 [0.14, 0.33]	0.21 [0.13, 0.32]	0.24 [0.15, 0.34]
Baseline HIV load, log10(copies/ml)	4.37 [3.81, 4.82]	4.41 [3.70, 4.93]	4.65 [4.19, 5.07]
Baseline WBC, 10^9/L	5.09 [4.26, 6.11]	5.01 [3.99, 5.90]	4.84 [4.06, 5.94]
Baseline HGB, g/L	144.00 [129.00, 153.00]	145.00 [129.00, 155.00]	142.00 [127.25, 153.00]
Baseline PLT, 10^9/L	208.00 [169.50, 250.00]	200.50 [167.00, 246.50]	198.00 [161.00, 237.00]

Baseline ALT, U/L	19.00 [14.00, 29.00]	19.00 [13.00, 30.25]	21.00 [15.00, 33.00]
Baseline AST, U/L	20.00 [17.00, 26.00]	21.00 [16.00, 28.00]	23.00 [19.00, 30.00]
Baseline TBIL, µmol/L	9.50 [7.00, 12.55]	9.80 [7.00, 13.00]	8.90 [6.40, 12.30]
Baseline CR, µmol/L	75.00 [66.00, 83.00]	77.50 [69.00, 87.00]	73.00 [65.00, 83.00]
Baseline TG, mmol/L	1.18 [0.88, 1.64]	1.12 [0.84, 1.56]	1.25 [0.87, 1.83]
Baseline CHOL, mmol/L	3.93 [3.50, 4.47]	3.99 [3.43, 4.53]	3.95 [3.46, 4.55]
Baseline FPG, mmol/L	5.07 [4.71, 5.43]	5.12 [4.77, 5.50]	5.10 [4.70, 5.60]

Abbreviations: IR, immune reconstitution; INR, immune non-responder; HAART, highly active antiretroviral therapy; INSTI, integrase strand transfer inhibitor; NNRTI, non-nucleoside reverse transcriptase inhibitor; PI, protease inhibitor; HBsAg, hepatitis B surface antigen; AntiHCV, anti-hepatitis C virus; WBC, white blood cells; HGB, hemoglobin; PLT, platelets; AST, aspartate aminotransferase; ALT, alanine aminotransferase; TBIL, total bilirubin; CR, creatinine; TG, triglycerides; CHOL, cholesterol; FPG, fasting plasma glucose.

Supplementary Figure 1. The changes in the clinical characteristics of PLWH within the external dataset across four follow-up points.

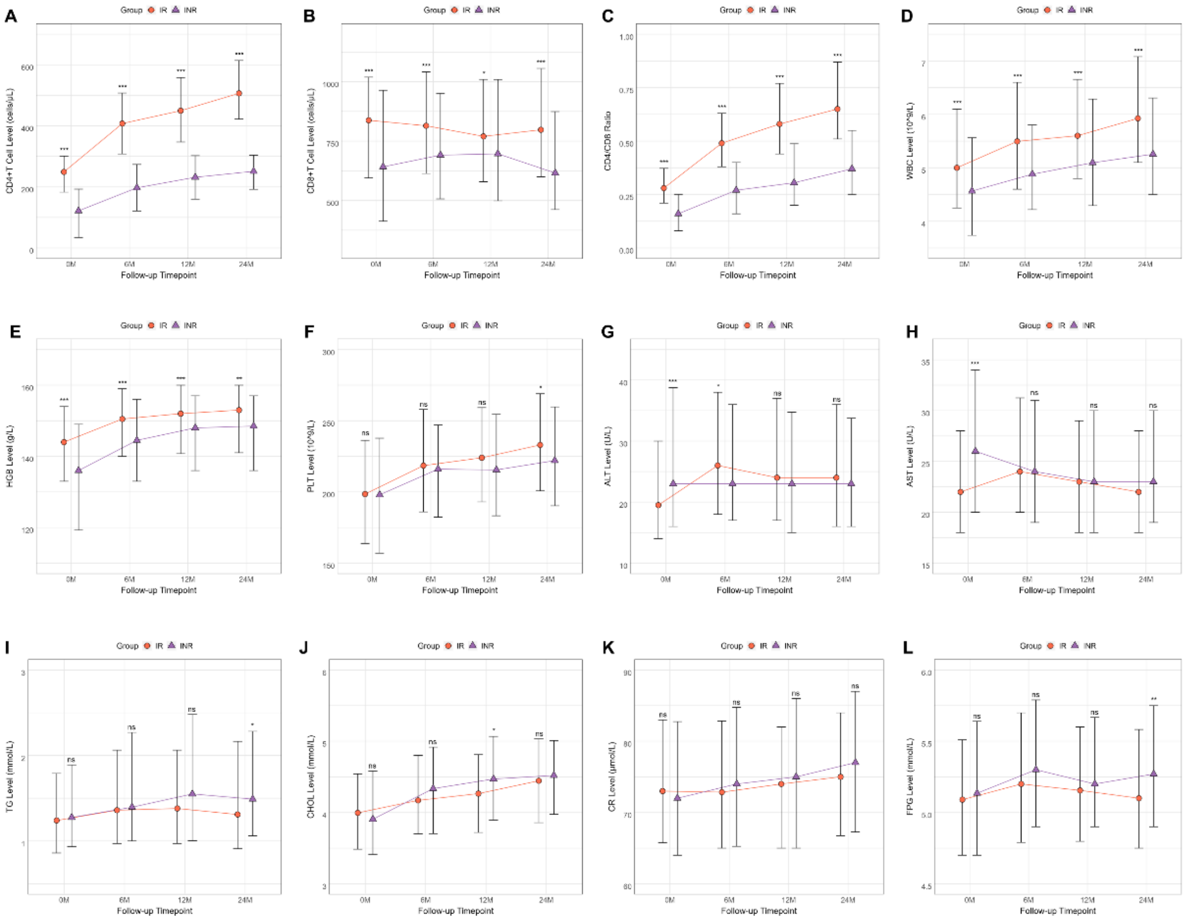

The changes in various parameters over different time-points during follow-up including CD4+T cells (A), CD8+T cells (B), CD4/CD8 ratio (C), WBC (D), HGB (E), PLT (F), ALT (G), AST (H), TG (I), CHOL (J), CR (K), and FPG (L).

Abbreviations: WBC, white blood cells; HGB, hemoglobin; PLT, platelets; ALT, alanine aminotransferase; AST, aspartate aminotransferase; TG, triglycerides; CHOL, cholesterol; CR, creatinine; FPG, fasting plasma glucose.

Supplementary Figure 2. Calculating variance inflation factors to assess multicollinearity among model variables.

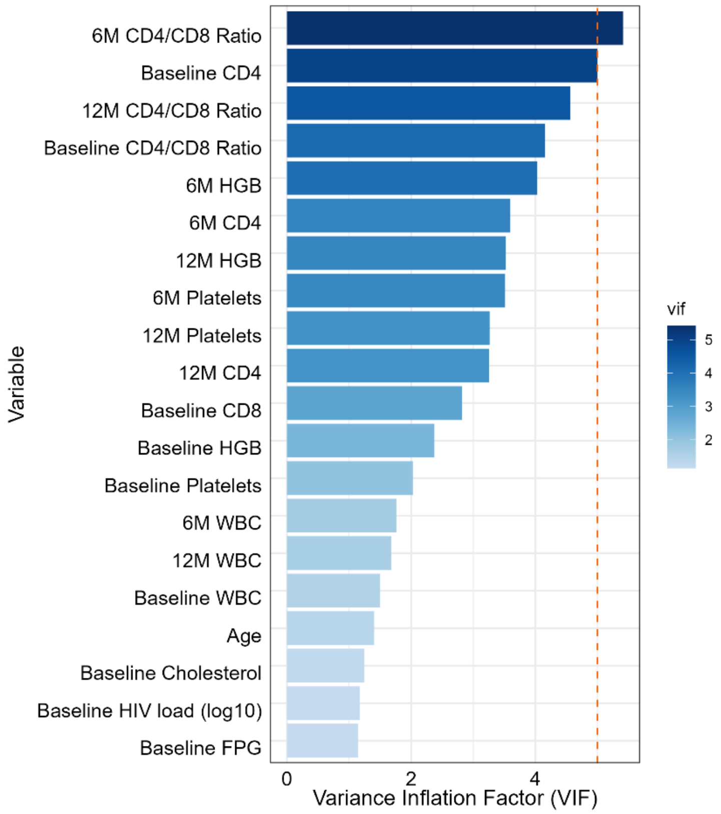

Abbreviations: WBC, white blood cells; HGB, hemoglobin; CHOL, cholesterol; FPG, fasting plasma glucose; PLT, platelets.

Supplementary Figure 3. Univariate and multivariate analysis of baseline parameters on INR

Characteristic	Univariate Regression OR (95% CI)	P value	Multivariate Regression OR (95% CI)	P value
Age	1.026 [1.015 - 1.038]	***<0.001***	1.021 [1.005 - 1.037]	***0.010***
Gender,Male	0.654 [0.415 - 1.029]	0.066		
Baseline-HIV Load(log10)	1.318 [1.110 - 1.570]	***0.002***	0.725 [0.574 - 0.911]	***0.009***
Baseline-HBsAg Positive	1.311 [0.881 - 1.947]	0.180		
Baseline-AntiHCV Positive	0.715 [0.150 - 2.727]	0.636		
Baseline-CD4	0.985 [0.983 - 0.987]	***<0.001***	0.983 [0.980 - 0.987]	***<0.001***
Baseline-CD8	0.999 [0.999 - 1.000]	***<0.001***	1.000 [1.000 - 1.001]	0.332
Baseline-CD4/CD8 Ratio	0.004 [0.001 - 0.013]	***<0.001***	0.699 [0.092 - 4.806]	0.722
Baseline-WBC	0.779 [0.707 - 0.857]	***<0.001***	0.842 [0.741 - 0.957]	***0.008***
Baseline-HGB	0.980 [0.974 - 0.987]	***<0.001***	1.012 [1.002 - 1.022]	***0.014***
Baseline-PLT	0.998 [0.996 - 1.000]	***0.032***	1.001 [0.998 - 1.004]	0.435
Baseline-ALT	1.000 [0.995 - 1.005]	0.913		
Baseline-AST	1.001 [0.996 - 1.006]	0.773		
Baseline-TBIL	0.980 [0.953 - 1.006]	0.134		
Baseline-CR	0.993 [0.983 - 1.002]	0.117		
Baseline-TG	0.908 [0.757 - 1.082]	0.287		
Baseline-CHOL	0.734 [0.619 - 0.865]	***<0.001***	0.959 [0.771 - 1.185]	0.700
Baseline-FPG	1.166 [1.023 - 1.343]	***0.025***	1.136 [0.959 - 1.358]	0.149

Axis: 0, 1, 2 — Low risk / High risk

Abbreviations: WBC, white blood cells; HGB, hemoglobin; PLT, platelets; ALT, alanine aminotransferase; AST, aspartate aminotransferase; TBIL, total bilirubin; CR, creatinine; TG, triglycerides; CHOL, cholesterol; FPG, fasting plasma glucose.
